# The human VGF-derived bioactive peptide TLQP-21 binds heat shock 71 kDa protein 8 (HSPA8)on the surface of SH-SY5Y cells

**DOI:** 10.1371/journal.pone.0185176

**Published:** 2017-09-21

**Authors:** Shamim Akhter, Sandipan Chakraborty, Daniela Moutinho, Elia Álvarez-Coiradas, Isaac Rosa, Juan Viñuela, Eduardo Domínguez, Angel García, Jesús R. Requena

**Affiliations:** 1 CIMUS Biomedical Research Institute, University of Santiago de Compostela-IDIS, Santiago de Compostela, Spain; 2 Biotechnology and Genetic Engineering Discipline, Khulna University, Khulna, Bangladesh; 3 Department of Microbiology, University of Calcutta, Kolkata, India; 4 BioFarma Research Group, CIMUS, University of Santiago de Compostela-IDIS, Santiago de Compostela, Spain; 5 Immunology Laboratory, Santiago University Hospital, Santiago de Compostela, Spain; University of Minnesota Twin Cities, UNITED STATES

## Abstract

VGF (non-acronymic)is a secreted chromogranin/secretogranin that gives rise to a number of bioactive peptides by a complex proteolysis mechanism. VGF-derived peptides exert an extensive array of biological effects in energy metabolism, mood regulation, pain, gastric secretion function, reproduction and, perhaps, cancer. It is therefore surprising that very little is known about receptors and binding partners of VGF-derived peptides and their downstream molecular mechanisms of action. Here, using affinity chromatography and mass spectrometry-based protein identification, we have identified the heat shock cognate 71 kDa protein A8 (HSPA8)as a binding partner of human TLQP-21 on the surface of human neuroblastomaSH-SY5Y cells. Binding of TLQP-21 to membrane associated HSPA8 in live SH-SY5Y cells was further supported by cross-linking to live cells. Interaction between HSPA8 and TLQP-21 was confirmed *in vitro* by label-free Dynamic Mass Redistribution (DMR) studies. Furthermore, molecular modeling studies show that TLQP-21 can be docked into the HSPA8 peptide binding pocket. Identification of HSPA8 as a cell surface binding partner of TLQP-21 opens new avenues to explore the molecular mechanisms of its physiological actions, and of pharmacological modulation thereof.

## Introduction

VGF is a neurotrophin-inducible neuropeptide precursor that in rodents has antidepressant-like actions [1–3] and regulates energy metabolism [4–5]. During the last few years, VGF has received increasing attention due to the progressive unveiling of its many physiological roles, especially in energy homeostasis and as a regulator of mood. Transgenic VGF ^+/−^ mice exhibit deficits in experimental paradigms of depression [1]. In turn, VGF^-/-^ mice are hypermetabolic and extremely lean [5].

VGF is a precursor of several bioactive peptides, which are generated by a process of proteolysis and are believed to mediate the effects of VGF [6–8]. Among these, TLQP-21 and TLQP-62 are of particular importance, and have been used in many *in vitro* and *in vivo* studies that have unveiled a wide range of effects mediated by these peptides [1,2,4,8–20]. In general, neurotrophic and mood-regulating effects have been ascribed to TLQP-62, while metabolic effects have been ascribed to TLQP-21; however, there seems to be an overlap of functions among products of VGF cleavage. Besides TLQP-21 and TLQP-62, other VGF-derived peptides like AQEE-30 and LQEQ-19 have been shown to elicit physiological effects [21].

TLQP-21 is a 21 residue peptide named after its four N-terminal amino acid residues [4]. TLQP-21 exerts multiple physiological roles, including modulation of energy expenditure, pain, gastric contractility and gastric acid secretion, reproduction, nociception, response to stress, lipolysis, and glucose-stimulated insulin secretion [4,7–9,10–20].TLQP-21 was also found to be a neuroprotective agent protecting rat cerebellar granule cells subjected to serum and potassium deprivation, via the modulation of extracellular signal-regulated kinase1/2, ERK1/2 and by the enhancement of intracellular Ca^2+^ release [21].

VGF and its derived peptides are also believed to play a role in cancer. Immunochemical studies have shown the presence of VGF immunoreactivity in most well-differentiated neuroendocrine tumors [22–24]. In particular, VGF is abundantly expressed in neuroblastoma cells[23,25–26] and recently we found that its expression in SH-SY5Y cells is regulated by DISC1, a protein encoded by a gene that has been associated to mental disease[26].

Remarkably, despite the large volume of data on effects of VGF-derived peptides, very little is known about their receptors, downstream signaling routes and mechanisms of action. Recently, complement component-3a receptor 1 (C3AR1)[27–28], and globular head of the complement component C1q receptor (gC1qR)[29], have been identified as potential receptors of murine TLQP-21. We reasoned that the neuroblastoma cell line SH-SY5Y would be a good model to study receptors and downstream mechanisms of VGF-derived peptides, given the substantial expression and secrete levels of VGF and VGF-derived peptides [23,25–26]. Such peptides might participate in autocrine processes, which should begin by their binding to receptors or binding partners present in the surface of these cells. We chose to work with TLQP-21 as more studies have focused on this peptide than on any other VGF-derived peptide.

Here we describe studies leading to the identification of heat shock cognate 71 kDa protein A8(HSPA8) as a binding partner of human TLQP-21 on cellular surfaces.

## Experimental procedures

### TLQP-21

TLQP-21 and amino-terminally biotinylated human TLQP-21 containing an extra cysteine residue at the C-terminus were purchased from ChinaPeptides (Wujiang, China); peptide purity, >95%, was confirmed by HPLC and MS analyses (data not shown).The peptide sequences are: TLQPPSALRRRHYHHALPPSR and biotin-TLQPPSALRRRHYHHALPPSRC, respectively. The lyophilized peptides were dissolved in filtered (0.22 μm, Millex, Merck Millipore Ltd.) phosphate buffered saline (PBS), pH = 7.4 and used instantly or kept at -80°C until used.

### SH-SY5Y cells

SH-SY5Y (European Collection of Cell Cultures, catalog number 94030304) cells were grown at 37°C in a 5% CO_2_-humidified incubator on the culture medium composed of 1:1 Earle’s Balanced Salt Solution (EBSS, Sigma Aldrich) and F12 HAM (Sigma Aldrich) supplemented with 15% fetal bovine serum (FBS, Gibco), 1% Glutamine (Gln), 1% Non-Essential Amino Acids (NEAA, Sigma Aldrich), and 1% Penicilin-Streptomicin (P/S, Invitrogen). Petri dishes with confluent SH-SY5Y cells were washed twice with cold PBS, followed by solubilization in lysis buffer, composed of 20 mM HEPES, 2mM EGTA, 1mM DTT, 1mM sodium orthovanadate, 1% Triton X-100, 10% Glycerol, 2μM leupeptin, 400 μM PMSF, 50 μM β-glycerophosphate, 100 μg/ml trasylol, pH = 8.1.The cells were scrapped on ice for 10 minutes and incubated on ice for 30 minutes with periodic vortexing every 5 minutes, followed by centrifugation for 20 minutes at 14000 g at 4°C. The supernatant was saved for further use. Protein concentration was quantified using the BCA protein assay kit (Pierce).

### Avidin-agarose affinity chromatography

A monomeric avidin column (Thermo Scientific) was washed with 8 ml PBS followed by addition of 6 ml (2 mM D-biotin in PBS, pH = 7.2) to block any non-reversible biotin binding sites. Regeneration buffer (0.1M glycine, pH 2.8, 12 ml) was added to remove biotin from the reversible binding sites followed by washing with 8 ml of PBS. One ml of 0.1 mM biotin-TLQP-21 was mixed with 900 μl of PBS and 100 μl of 1mM TCEP and the resulting solution was applied to the column, which was incubated for an hour at room temperature. The column was then washed with 12 ml of PBS. A 2ml solution containing 700 μl of SH-SY5Y cell lysate with a protein concentration of 10μg/μl was diluted with 1.3 ml of PBS and applied then to the column. The column was then washed with 12 ml of PBS, and eluted with 10 ml of blocking/elution buffer. Fractions of 500 μl were collected and kept on ice until further use for analysis by SYPRO^®^ Ruby Protein Gel Stain (Lonza), and Western blot. As a control, cell lysate solution was added under the same conditions to a column to which biotin-TLQP-21 was not attached.

### SDS-PAGE and SYPRO Ruby protein gel stain

Portions (25 μl) of each elution fraction from avidin-agarose affinity chromatography were boiled in 2x Laemmli buffer and loaded to a 10% SDS-PAGE gel. Electrophoresis was carried out at 200V for 60 minutes. After completion, the gel was washed in dH_2_O for 10 minutes. Then the gel was incubated with fixing solution (10% methanol, 7% acetic acid) for 1 hour at room temperature in an orbital shaker set at 50 rpm, followed by overnight incubation with SYPRO Ruby protein gel stain at room temperature with shaking and protection from light. The gel was then transferred to a clean staining container, and washed for 5 minutes with fixing solution followed by washing for 5 minutes in dH_2_O.Finally, the gel was imaged in a Molecular Imager Gel Doc system (Bio Rad, Hercules) using the highest sensitivity of the CCD camera (CoolSNAP HQ2, Roper Scientific) at a resolution of 1392 x 1040 pixels with 12 bit gray scale levels per pixel.

### Protein identification

Protein bands of interest detected after SDS-PAGE of affinity chromatography fractions were excised and subjected to in gel reduction, alkylation and trypsin digestion [30]. Extracted peptides were analyzed by LC-MS/MS. Briefly, digested peptide mixtures were dried in a centrifugal evaporator (Speedvac, Savant) and dissolved in 0.1% formic acid followed by separation in an EASY-nLC (Proxeon, Bruker Daltonik GmbH) with a reverse phase nanocolumn (Easy column SC200 C18 3μm 120A 360 μm OD/75μm ID, L = 10cm) from Proxeon. Ionized peptides were detected in a Bruker Amazon ETD ion trap. Automated analysis of mass data was performed by Data Analysis 4.0 and BioTools 3.2 from Bruker Daltonik GmbH. Database search was done with the Mascot v2.3 search tool (Matrix Science) screening SwissProt (release 57.15). Searches were limited to human taxonomy selecting carbamidomethyl cysteine as a fixed modification and oxidized methionine as potential variable modification. Both the precursor mass tolerance and the MS/MS tolerance were fixed at 0.3 and 0.4 Da, respectively, with 1 missed tryptic cleavage site. All spectra and database results were scrutinized manually in details using the above software, especially in the case of identifications based on one peptide hit. Positive identification by MS was only accepted when more than 50% y-ions were obtained for a peptide comprising at least eight amino acids long and no missed tryptic cleavage site. Positive hits corresponded to Mascot scores > 40 plus the fulfillment of the above criteria.

### Immunoassays

After electrophoresis, as described earlier, proteins were transferred onto PVDF membranes (Millipore, Bedford) using semi-dry method (Trans-blot SD semi-dry transfer cell, Biorad). The membranes were then blocked 5% BSA in TBS-T solution (Tris-buffered saline with 0.1% tween 20, pH = 7.6) for 1 hour at room temperature, and probed with anti-HSC70/HSPA8 (Abcam) antibody at 1:500 dilution. Anti-rabbit antibody (Dako) at a 1:2000 dilution was used as the secondary antibody. Subsequently, the membrane was incubated with Luminata Forte Western HRP substrate [Merck Millipore] for 5 minutes and developed using Hypercassette and Amersham hyperfilm (GE Healthcare).

### Crosslinking of biotinylated TLQP-21 to SH-SY5Y surface membrane proteins

SH-SY5Y cells were grown as described above until confluence. Culture medium was removed, cells were washed three times with cold PBS and incubated with 2 mL of PBS containing 1 mM Sulfo-EMCS with 50 μM biotin-TLQP-21 for 1 hour at room temperature. Control cells were incubated under the same conditions with the same reaction mixture lacking biotin-TLQP-21. The cross-linking reaction was stopped adding Tris-HCl, pH 7.8 to a final concentration 50 mM. Cells were washed twice with cold PBS and solubilized in lysis buffer (20 mM HEPES pH 7.4, 2 mM EGTA, 1 mM DTT, 1 mM sodium orthovanadate, 1% Triton X-100, 10% glycerol, 2 μM leupeptin, 400 μM PMSF, 50 μM β-glycerophosphate and 100 μg/ml aprotinin). The cells were scrapped on ice for ten minutes and incubated on ice for 30 minutes with periodic vortexing at each 5 minutes, followed by centrifugation at 4°C, 14000 g for 20 minutes. The supernatant, containing the cell membrane fraction, was recovered and further centrifuged at 4°C, 50 000 g for1h. The resulting membrane pellet was resuspended in PBS with 1 mM DTT and 2% n-octyl-β-D-glucopyranoside for membrane protein solubilization, and briefly sonicated. Two ml of solubilized biotinylated SH-SY5Y membrane proteins were applied to the Pierce avidin-agarose affinity column, previously conditioned by sequential washes with 8 ml PBS, 6 ml of blocking/elution buffer, 12 ml of regeneration buffer and finally, 8 ml of PBS, according to the manufacturer´s instructions. The column was then washed with 12 ml of PBS, and eluted with 10 ml of blocking/elution buffer. The elution fraction was collected and stored on ice for further precipitation in 85% cold methanol, resuspended in PBS with 1 mM DTT and subsequently subjected to SDS-PAGE and Western blotting as described above.

### Analysis of protein-peptide interaction by label-free Dynamic Mass Redistribution (DMR) technology

Label-free high sensitivity plates (PerkinElmer 6057460) were activated with 15 μl of 400 mM N-(3-dimethylaminopropyl)-N’-ethylcarbodiimide hydrochloride (EDC) (Sigma-Aldrich) and 100 mM Sulfo-N-hydroxysulfosuccidime (sulfo-NHS) (ThermoFisher) diluted in ultrapure water 30 min at room temperature. Microplates were subsequently washed four times with ultrapure water.

HSPA8 immobilization was performed by adding 15 μl of 25 μg/ml of protein in 20 mM sodium acetate buffer at pH = 5. After overnight incubation at 4°C, microplates were washed four times with PBS containing 0,005% Tween-20 buffer, pH = 7.4. Baseline was read after the microplate was equilibrated inside the EnSpire® Multimode Plate Reader (Perkin Elmer) for 3 hours.

TLQP-21 peptide dilutions were prepared in PBS containing 0,005% Tween-20 buffer, pH = 7.4. To this, 15 μl of peptide solution were added to the plate and mixed. Final reading was performed every two minutes over a period of 1 hour.

### *In silico* molecular modeling and docking study

The complete structure of the human HSPA8 is not available and in most of the available partial crystal structures of the protein, the lid domain moves over the substrate binding cavity leading to cavity closure. This conformation does not allow the substrate binding. Thus, to probe TLQP-21 binding to HSPA8, we need to model HSPA8 in open conformation where the lid is further away from the substrate binding domain. Therefore, we modeled the HSPA8 in open conformation using its *E*. *coli* homolog Hsp70 DnaK (PDB id: 4JNE). The x-ray crystal structure of *E*. *Coli* DnaK has been solved at a resolution of 1.96 Å where the lid domain orients away from the substrate binding domain makes the substrate recognition site wide open, thus allowing substrate binding. Sequence analysis reveals that the human HSPA8 shares 52.82% sequence identity with *E*. *Coli*Hsp70 DnaK. Therefore, we used homology modeling to model the HSPA8 in open conformation using the SWISS-MODEL web-server [31] and the quality of the generated model was judged using ANOLEA and QMEAN analysis. The modeled HSPA8 was optimized using the GROMACS 4.5 packages [32–33] with OPLS force field [34].The structure of the modelled protein was refined through500 steps energy minimization using the steepest descent algorithm *in vacuo*. The vacuum minimized structure was further minimized by 500 steps using steepest descent algorithm followed by 1500 steps of conjugate gradient energy minimization in water using TIP3P explicit water model in a cubic box with periodic boundary condition.

Secondary structure analysis of human TLQP-21 (TLQPPSALRRRHYHHALPPSR) from its amino acid sequence using Jpred3 web-interface [35–36] reveals strong helical propensity in the region 6–13. Also, recent NMR study by Cero et al. demonstrated that TLQP-21 peptide adopts a well-defined α-helical conformation in the presence of 3T3L1 cells expressing a putative receptor of the peptide [28]. Therefore, the peptide was modeled as being α-helical considering both the C and N terminal amino and carboxylic groups in neutral form. The modeled TLQP-21 peptide was also optimized using the GROMACS 4.5 packages [32–33] with OPLS force field [34].The peptide was subjected to a short 100 steps energy minimization using the steepest descent algorithm *in vacuo* which is followed by 3000 steps of *in vacuo* conjugate gradient minimization. Then the minimized peptide was solvated with TIP3P explicit water model in a cubic box with periodic boundary condition. The solvated system was then subjected to 500 steps of energy minimization using steepest descent algorithm in water.

Protein-peptide docking was used to dock the TLQP-21 within HSPA8 substrate binding site. Initially, we used ClusPro 2.0 web-interface [37–38] to dock the TLQP-21 with HSPA8. ClusPro uses a fast Fourier transform (FFT) based rigid body docking algorithm to explore all possible binding possibilities of the peptide within the active site by a systematic rotation and translation of the peptide. Then 1000 lowest energy solution judged by a balanced scoring function are clustered and solutions from the 10 best clusters are reported. The best solution was selected which has the most favorable energy and also belongs to the most populated cluster.

### Molecular dynamics simulation of the HSPA8-TLQP-21 complex

Conformational dynamics of theHSPA8-TLQP-21complex were studied using extensive molecular dynamics simulation with the aid of GROMACS 4.5 packages [32–33] with OPLS force field [34].The complex was subjected to a preliminary short energy minimization *in vacuo* using the steepest descent algorithm. Then the system was solvated with TIP3P explicit water model in a cubic box with periodic boundary condition. The box dimension was chosen such that all the protein atoms were at a distance equal to or greater than 10 Å from the box edges. The simulated system was then made charge neutral by adding appropriate number of counter ions. The solvated system was then subjected to 1000 steps of energy minimization using steepest descent algorithm which was followed by 5000 steps of minimization using conjugate gradient algorithm. After that, a 100 ps position restrained dynamics was carried out where the complex was restrained by adding restraining forces while the water molecules were allowed to move freely. It allows proper equilibration of solvent around the solute. It was then followed by 100 ps of unrestrained NVT simulation at 300 K and followed by 100 ps of NPT simulation to achieve proper equilibration of the system to be simulated. Final production simulations were performed in the isothermal isobaric (NPT) ensemble at 300 K, using an external bath with a coupling constant of 0.1 ps. The pressure was kept constant (1 bar) by using pressure coupling with the time-constant set to 1 ps. The LINCS algorithm was used to constrain the bond lengths involving hydrogen atoms, allowing the use of 2.0 fs time step. Electrostatic interactions were calculated using particle mesh Ewald summation method. Van der Waals interactions were truncated at 14 Å. The trajectories were stored at every 5 ps. Analyses were carried out with the trajectory analysis tools implemented in GROMACS and the secondary structure assignments were carried out with DSSP [39] module. Also, a 100 ns simulation of free TLQP-21 in explicit water was also carried out using GROMACS 4.5 [32–33] packages with OPLS force field [34]. Details of the simulation methodology used are in accordance to our recently published paper [40].

## Results

### Identification of a human TLQP-21 surface binding protein

**Fig 1:**
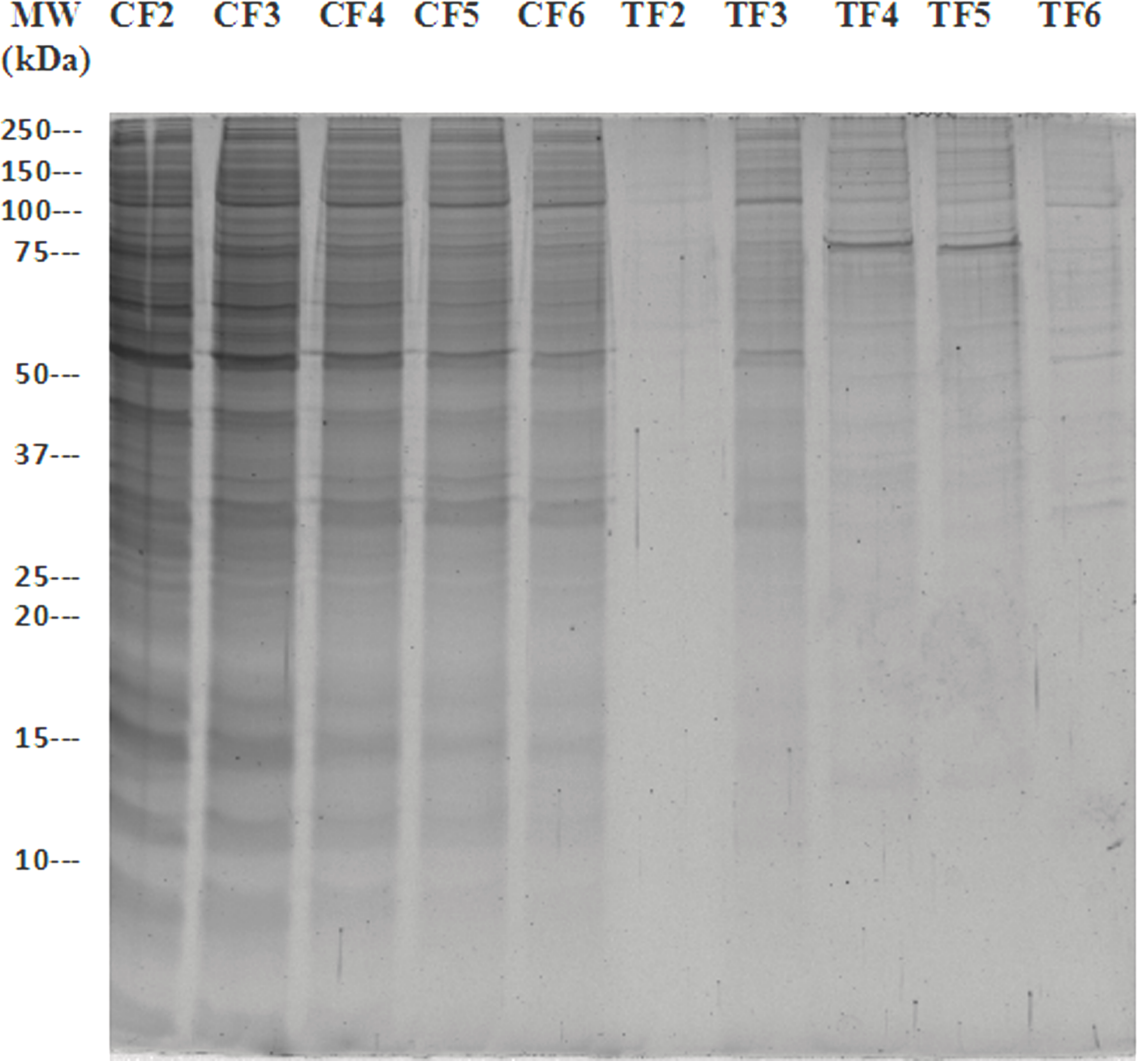
Identification of a TLQP-21 binding protein from SH-SY5Y cell homogenates. A ~70 kDa protein was bound on the biotin-TLQP-21 treated/attached column (fractions TF 4–5) but not in the corresponding fractions eluted from the control column (*i*.*e*., from a sample prepared in the same way but subjected to chromatography on an affinity column which had not been pre-loaded with biotin-TLQP-21:CF4-5). The eluted fractions were resolved by SDS-PAGE and analysed by SYPRO^®^ Ruby Protein Gel Stain.

Following chromatography of SH-SY5Y cell homogenates on avidin agarose affinity chromatography [29], and SDS-PAGE of the column eluate, a protein band of ∼70 kDa was detected by SYPRO Ruby staining. The band was not present in the eluate from the control sample applied to an avidin agarose column lacking the biotinyl-TLQP-21 peptide (Fig 1). This band was excised and analyzed by nano- HPLC-electrospray MS/MS. MASCOT analysis of the spectra (Table 1) identified a total of 3 proteins, considering exclusion parameters [41]: Heat shock cognate 71 kDa protein (HSPA8/HSC70), Stress-70 protein (HSPA9/mortalin), and 78 kDa glucose-regulated protein (HSPA5). Sequence coverages and scores varied from highest for HSPA8 (19.8, 735), middle for HSPA9 (12.5, 431), to lowest for HSPA5 (5.4, 216). For HSPA5, only 3 peptides were identified, at the borderline of exclusion parameters; therefore, we concentrated our attention on HSPA9 and, in particular, HSPA8.

**Table 1:** MASCOT analysis of proteins present in the ~70kDa band detected after affinity chromatography of SH-SY5Y cell homogenates using TLQP-21 as a “bait”.

Protein	Score	MW (kDa)	Sequence coverage	Number of unique peptides
Heat shock cognate 71 kDaprotein(HSPA8/Hsc71)	735.0	70.9	19.8	12
Stress-70 protein (HSPA9/Mortalin)	430.0	73.6	12.5	07
78 kDa glucose-regulated protein(HSPA5/GRP78)	216.0	72.3	5.4	03

### Immunochemical validation of HSPA8

**Fig 2:**
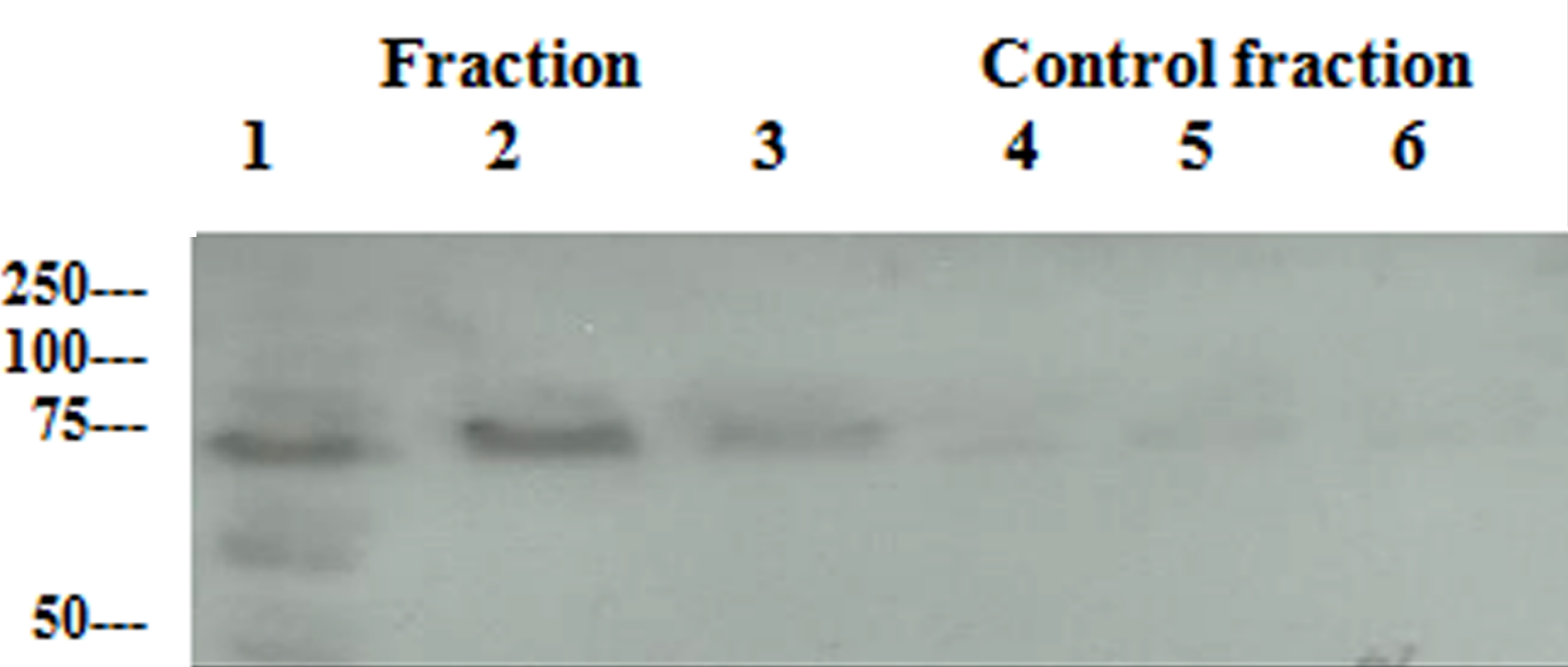
Western blot, using an anti-HSPA8 antibody, confirmed the TLQP-21 binding protein from SH-SY5Y cell homogenates as HSPA8. Western blot analysis of fractions eluted from the biotin-TLQP-21-containing column confirmed the ~70 kDa protein as HSPA8 but not in the controls. Elution fractions 4, 5 with corresponding control fractions 4, 5 were pooled together (see Fig 1) and used with three replicates. Lanes 1–3: treated fractions, from biotin-TLQP-21 treated /attached column, Lanes 4–6: control fractions, *i*.*e*., from a sample prepared in the same way but subjected to chromatography on an affinity column which had not been pre-loaded with biotin-TLQP-21.

The samples corresponding to fractions 4 and 5 of the immunoaffinity column eluate (Fig 1)in which the ~70kDa band detected by SYPRO^®^ Ruby protein gel stain, were pooled together and subjected to Western blot analysis with an anti-HSC70 (HSPA8) antibody. As seen in Fig 2, the antibody reacted positively with the protein but did not bind to lanes corresponding to equivalent control fractions.

### Binding of biotinylated TLQP-21 to HSPA8 on the SH-SY5Y membrane

**Fig 3:**
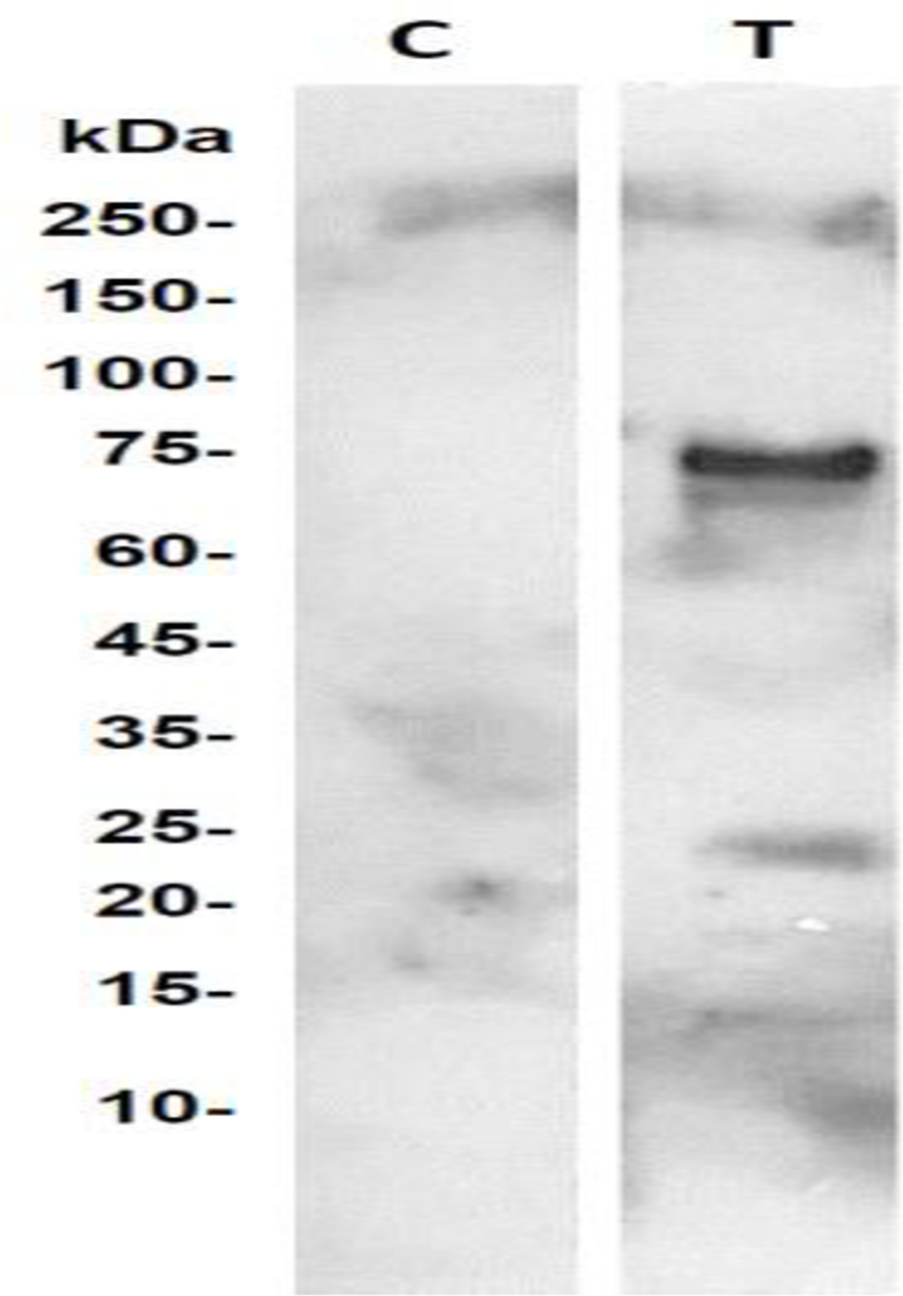
TLQP-21 binds to HSPA8 expressed on the cell surface of live SH-SY5Y cells. Membrane proteins, following biotinylation, were applied onto the monomeric avidin column. The eluted proteins (T) were analysed by the Western blot using anti-HSPA8 antibody; the control sample (C) was obtained from cells treated under the same conditions, but no TLQP-21 was added.

Given that HSPA8 is distributed not only in the cell membrane [42] but also in the cytoplasm [43], and that we had used a whole cell homogenate in our experiment to trap TLQP-21 binding partners, we sought to confirm that TLQP-21 binds to HSPA8 located in the cell membrane. For this, biotinylated TLQP-21 was added to intact, live SH-SY5Ycells and cross-linked to accessible membrane binding partners with the bifunctional crosslinking reagent sulfo-EMCS. Subsequently, the cells were homogenized and fractionated, and the membrane fraction subjected to affinity chromatography using a monomeric avidin column. Chromatographic fractions eluted from the column were resolved by SDS-PAGE, blotted, and membranes probed with anti-HSPA8 antibody. A positive ~70kDa band corresponding to HSPA8 was detected in samples from cells treated with TLQP-21 under surface protein-cross-linking conditions, but not in control samples (Fig 3).

### Characterization of the TLQP-21 binding to HSPA8 using Dynamic Mass Redistribution analysis

**Fig 4:**
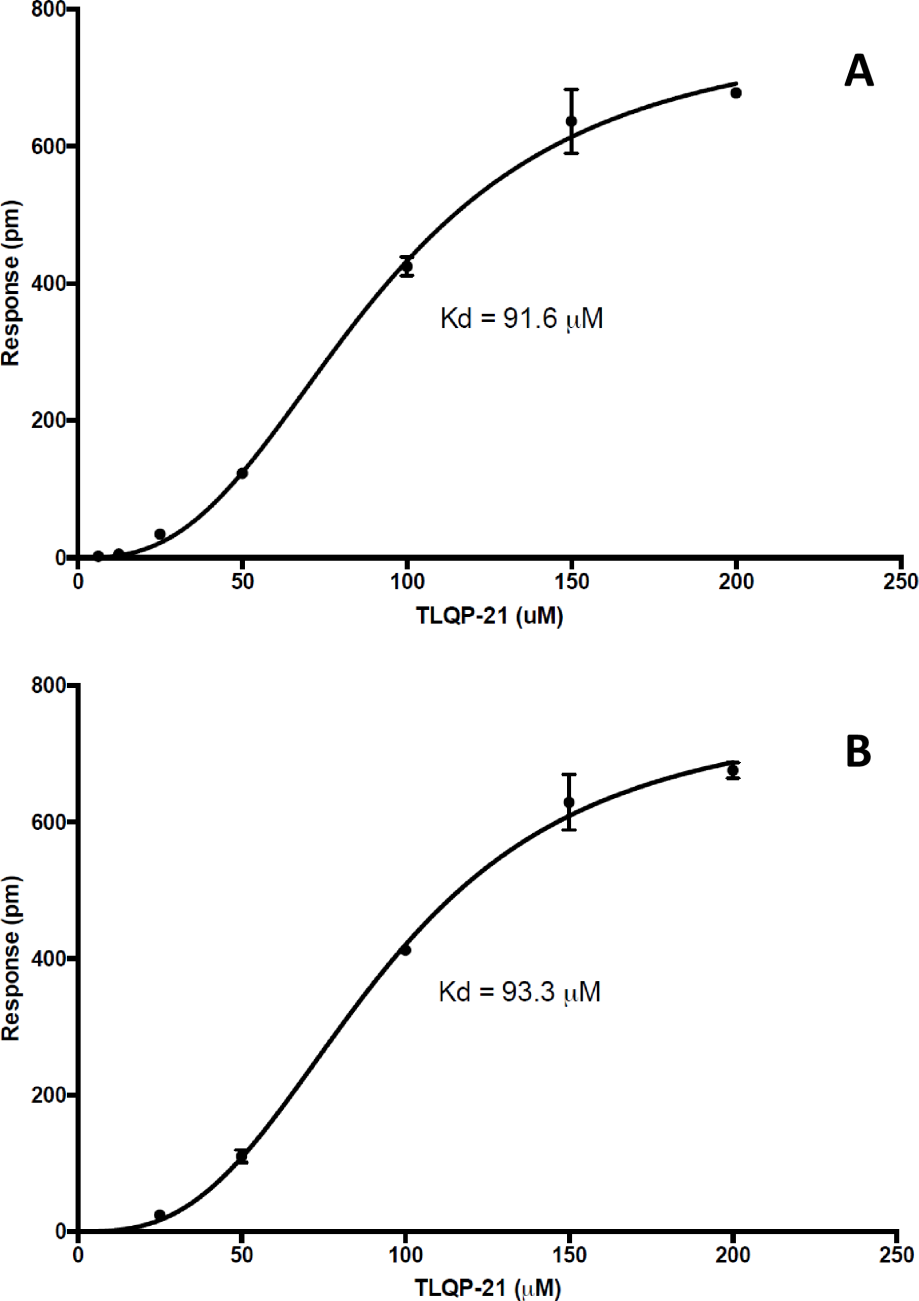
Dynamic Mass Redistribution analysis of TLQP-21 interaction with HSPA8. A range of different concentrations of peptide were tested (6.25, 12.5, 25, 50, 100, 150 and 200 μM) in triplicates. Responses are shown at 20 (A) and 60 (B) minutes obtaining an K_d_ of 91.56 and 93.26 μM, respectively. Label-free responses are measured as shifts in reflected wavelength and are expressed in picometers (pm).

Different concentrations of TLQP-21 peptide (6.25, 12.5, 25, 50, 100, 150 and 200 μM) were tested for their ability to bind HSPA8 immobilized on a DMR plate. Responses at 20 and 60 minutes are shown in Fig 4. Robust binding was confirmed at both times, with calculated K_d_ of 91.56 and 93.26 μM, respectively.

### Modelling recognition of TLQP-21 by HSPA8 *in silico*

**Fig 5:**
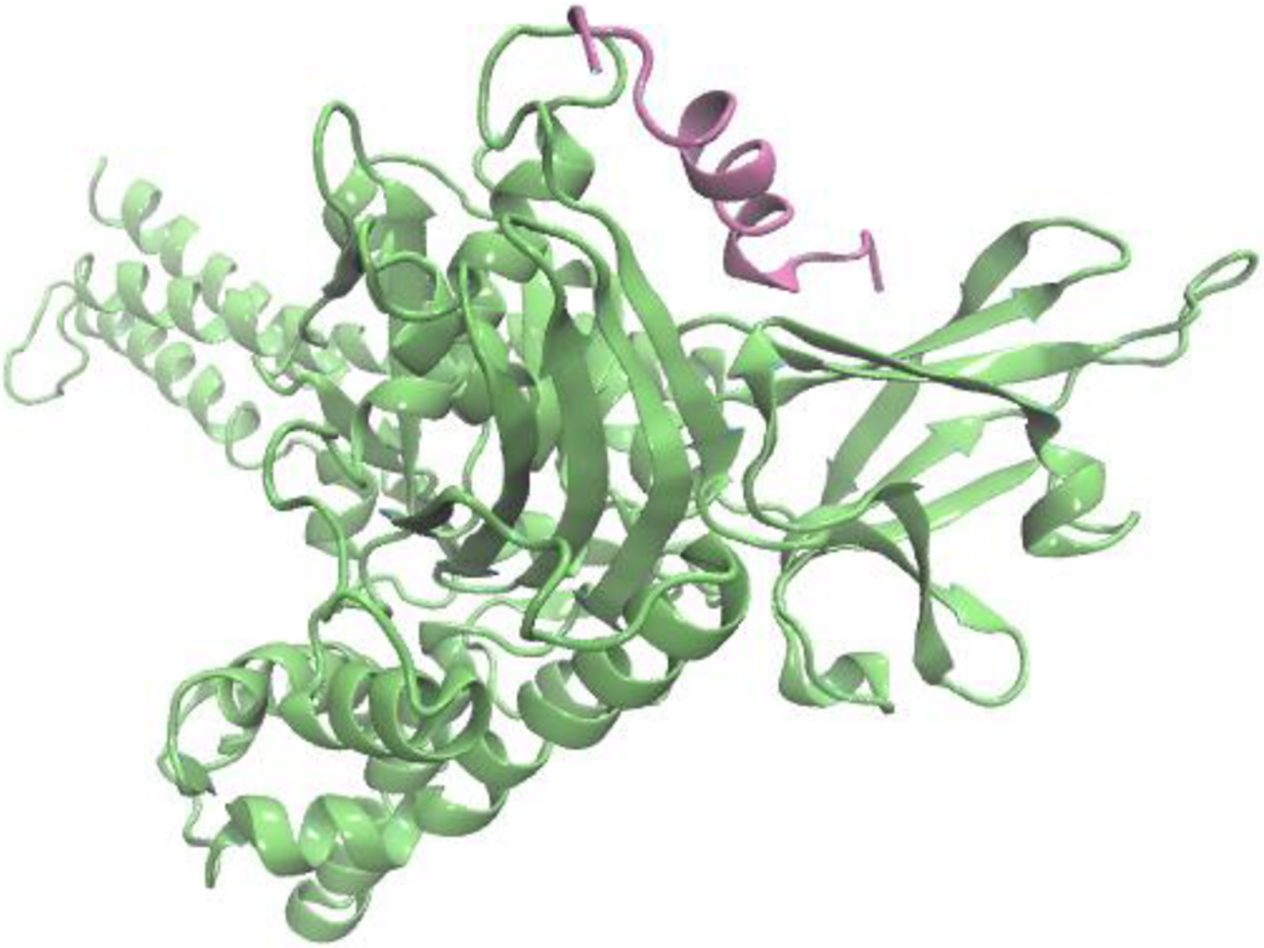
Structures of the HSPA8-TLQP-21 obtained from molecular docking study. HSPA8 is colored in green whereas the bound TLQP-21 is colored in pink.

To explore the recognition mechanism of TLQP-21 by HSPA8, we employed protein-peptide docking implemented in ClusPro 2.0 web-interface. It is to be noted that we have modeled the HSPA8 in open conformation where lid is further away from the substrate binding domain, as observed in its prokaryotic homolog *E*. *coli* Hsp70 DnaK. In this lowest energy docking complex TLQP-21 binds properly within the substrate binding domain of HSPA8. The structure of the TLQP-21-HSPA8 complex obtained from ClusPro is shown in Fig 5.

As evident from the figure, TLQP-21 appropriately orients within the substrate binding site of HSPA8. The peptide interacts strongly with the twisted β-sheeted plate sheet region of the HSPA8 substrate binding site. The complex displays high steric compatibility in size and shape between the TLQP-21 and HSPA8, resulting a tighter spatial fit of the peptide inside the cavity.

### Molecular dynamics simulation of the complex

**Fig 6:**
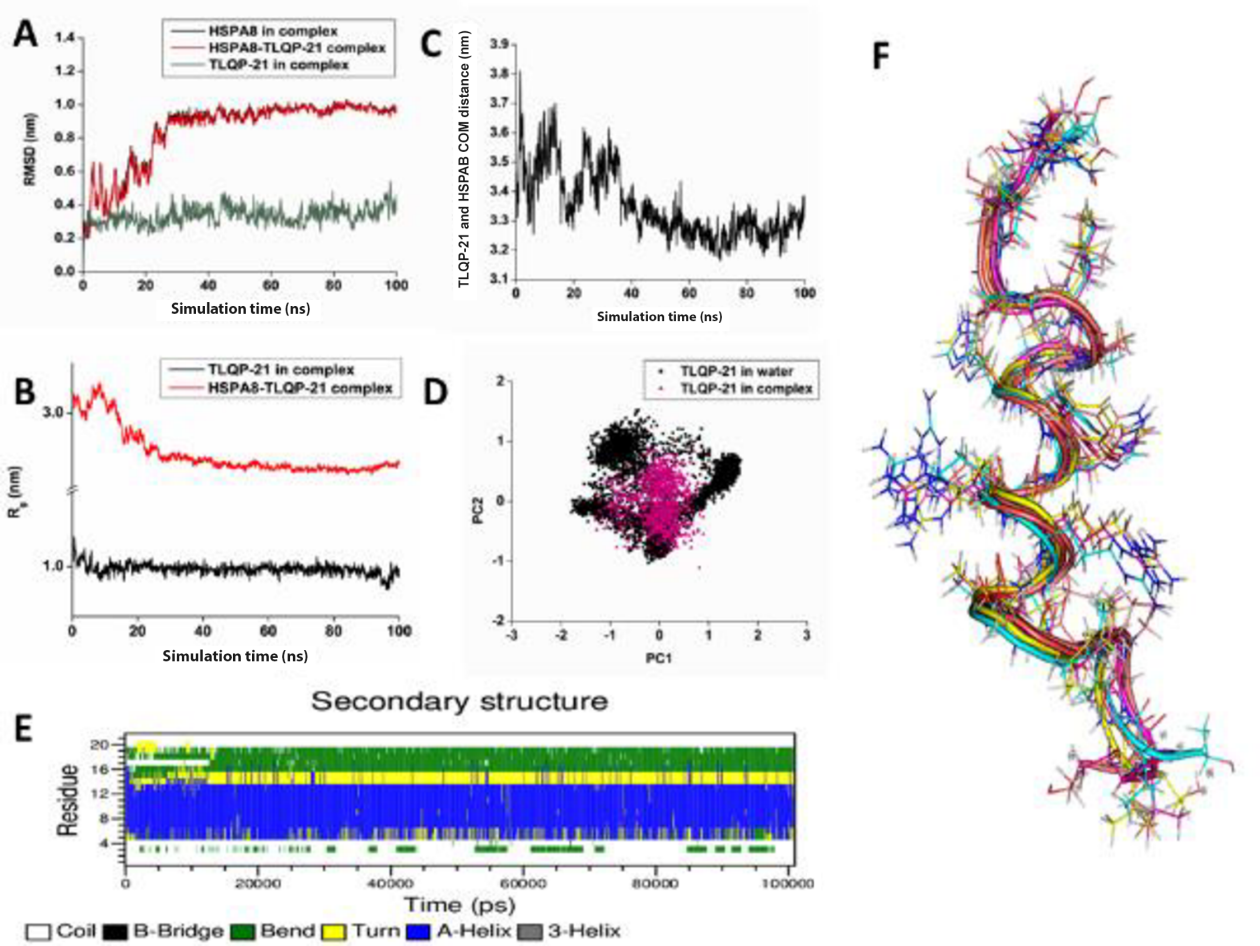
**Variations of RMSD (A) and R**_**g**_ **(B) with simulation time for HSPA8-TLQP-21 complex in solution obtained from molecular dynamics simulation**. C: Changes of the center of mass (COM) distance between HSPA8 and TLQP-21 in the complex during simulation time. D: Principal Component Analysis obtained from MD simulations. 2-D projection of the TLQP-21 in free (black) and bound state (pink) in bi-dimensional subspace defined by the first two PCs (PC1 and PC2) on the simulation trajectories is shown. E: Secondary structural propensity variation analysis of each residue of the peptide with simulation time when complexed with HSPA8. F: Superimposed structure of the five most populated conformations of the peptide TLQP-21 obtained from cluster analysis of simulation trajectories.

Stability of the HSPA8-TLQP-21 complex and conformational dynamics of TLQP-21 in HSPA8 bound state was assessed using molecular dynamics (MD) simulation using GROMACS packages [32–33]. Fig 6A and 6B depict the variations of two global structural parameters root mean square deviation (RMSD) and radius of gyration (R_g_) of the TLQP-21 in bound state. Fig 6A shows that the complex undergoes significant conformational changes during the first 25 ns of the simulation, after that the system monotonically reaches to an equilibrium state, evident from the stable RMSD profile of the complex. We then analyzed the variations of the RMSD of each of the component of the complex, i.e., HSPA8 and TLQP-21, during the simulation. As evident from the Fig 6A, changes observed in the complex during the initial period of the simulation is mainly contributed by HSPA8. Interestingly, the TLQP-21 in complex with HSPA8 is highly stable throughout the simulation. The initial helical conformation of the peptide undergoes very small changes in its RMSD (~ 0.3 nm with respect to initial conformation) during initial equilibration period after that it remains stable throughout the remaining simulation period. On the other hand, radius of gyration (R_g_) provides insight into the overall dimension and shape of the simulated system. The initial structure of the complex changes rapidly until ~25 ns and adopts a more compact form which remains stable in remaining simulation time (Fig 6B). In contrast, the initial helical conformation of the peptide TLQP-21, when bound to HSPA8, is highly stable during the 100 ns simulation of the complex, evident from a highly stable R_g_ profile (Fig 6B). In a recent study, we have explored the conformational landscape of the free TLQP-21 peptide in solution using 550 ns MD simulation using the same protocol used in this study. The initial long helical conformation is not stable and the peptide refolds in a more compact globular form [40].Thus molecular dynamics simulations reveal that the peptide in free form populates several states, while the bound form is relatively stable. Our simulation data reveals that TLQP-21 adopts a defined α-helical form only after binding to HSPA8. Thus, the peptide undergoes refolding after binding.

Analysis of the center of mass (COM) distance variations between TLQP-21 and HSPA8 reveals that the peptide TLQP-21 remains attached with HSPA8 throughout the simulation and never dissociates (Fig 6C). During the initial period of the simulation where the complex reorients it according to its environments, evident from the RMSD and R_g_ changes, the COM distance varies and stabilized when the complex is properly equilibrated after 30 ns of the simulation. In fact, the initial COM distance between TLQP-21 and HSPA8 is 3.45 nm in the docked complex decreases during the equilibration period it stabilized ~3.30 nm during the last 60 ns of the simulation. Thus during the simulation TLQP-21 comes closer and binds strongly to the HSPA8 binding site (Fig 6C).

Principal component analysis (PCA) is an efficient data reduction technique where the dimensionality of the essential subspace of the peptide is defined by the reduced subspace represented by eigenvectors or principal components (PCs). The first few components are more informative as evident with high eigenvalues while the latter components carry very little information about the peptide dynamics. Comparisons of conformational landscape of the free and bound peptide in essential subspace have been represented in terms of the first two PCs (PC1 and PC2) and are shown in Fig 6D. Here, each point in the essential sub-space defines a unique conformation of the simulated system and structurally similar conformations are clubbed together in the essential sub-space. The free TLQP-21 peptide is highly flexible during the simulation and visits numerous distinct conformational states as evident from a widespread projection distribution in essential space constituted by first two PCs while upon binding, the flexibility of the peptide is greatly reduced evident from the highly reduced spatial distribution in essential subspace.

Secondary structural propensity analysis of each residue of the TLQP-21 peptide when bound with HSPA8 throughout the simulation time is shown in Fig 6E. TLQP-21 when bound to the HSPA8, the peptide possess strong helical propensity and it’s helical conformation is highly stable throughout the simulation. Particularly, the region 4–14 exists as α-helix when bound to its binding site in HSPA8. Interestingly, our recent simulation study shows that TLQP-21 in solution the peptide is highly flexible and mostly unstructured. Most of the residues of the peptide possess high turn/bend propensity with very little helical propensity [40].

To identify most frequently visited conformations during MD simulation, cluster analysis has been performed over the 100 ns trajectory using a stringent RMSD cutoff of 0.1 nm using GROMOS algorithm. Average conformation of the five most populated clusters of TLQP-21 when bound to HSPA8, observed during the simulation, is shown in Fig 6F. All the five conformations of the peptide have very similar side-chain orientations and exist as long α-helix.

**Fig 7:**
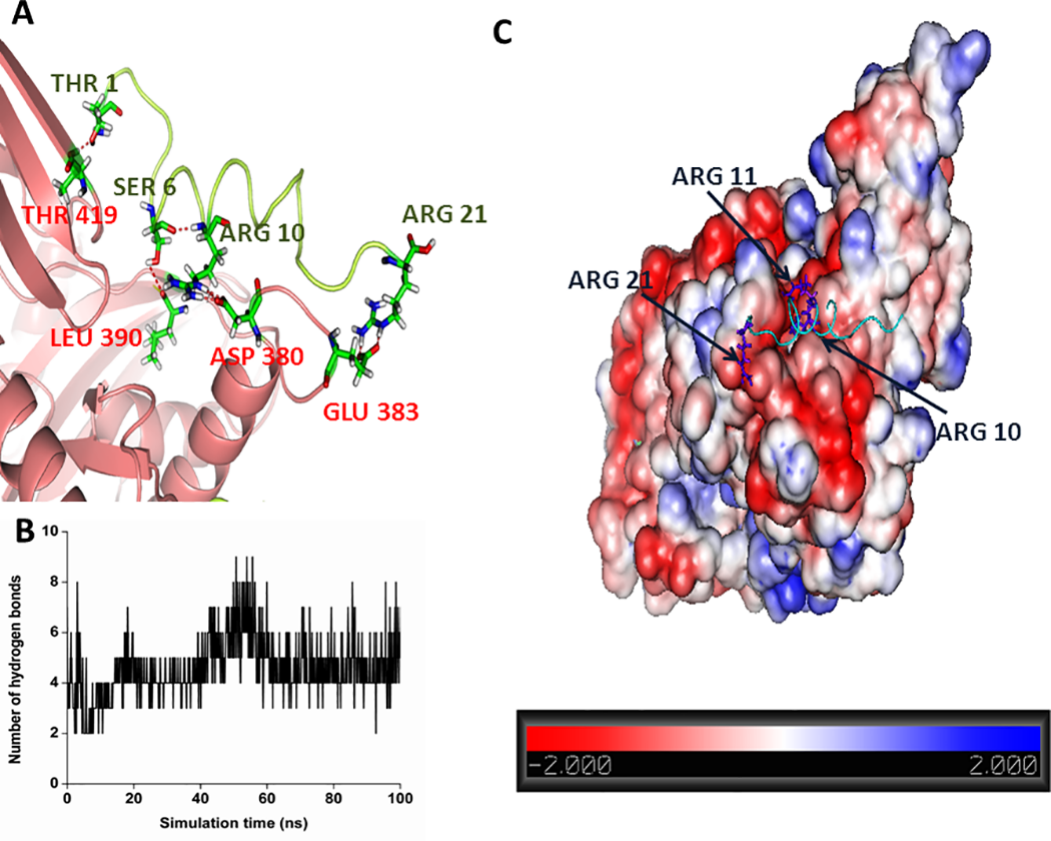
Characteristics of the interactions involved in TLQP-21 recognition by HSPA8. A: Details of the hydrogen bonding interactions between TLQP-21 and HSPA8. HSPA8 and TLQP-21 are colored as salmon and lime-green color, respectively. B: Variations of the number of hydrogen bonding between TLQP-21 and HSPA8 with simulation time. C: Electrostatic surface potential of HSPA8 when TLQP-21 is bound to the protein. Regions of positively and negatively charged surface area are colored in blue and red color, respectively. Arginine residues of TLQP-21 are shown in blue stick mode.

We also analyzed the hydrogen bonding interactions between TLQP-21 and HSPA8 in the most populated complex structure observed in the MD simulation using PIC web-server [44] and are shown in Fig 7A. Thr1 at the N-terminal of the TLQP-21 is involved in hydrogen bonding interaction with Th 419 of HSPA8. At the middle of the peptide, Ser6 and Arg 10 are involved in hydrogen bonding interactions with Leu 390 and Asp 380, respectively at the HSPA8 peptide binding site. These interactions might play crucial role in maintaining the helical structure of the peptide within its binding site. At the C-terminal region, Arg 21 of TLQP-21 is involved in a hydrogen bonding interaction with Glu383 of HSPA8. Fig 7B demonstrates the variation of number of hydrogen bonds between TLQP-21 and HSPA8 during the simulation. As evident from the figure, during the initial 50 ns of the simulation the number of hydrogen bonds varies greatly since the complex undergoes conformational readjustment, as evident RMSD and R_g_ plot. During latter half of the simulation where the complex is well equilibrated, the observed hydrogen bonds remain stable throughout the simulation. Fig 7C shows electrostatic interactions between HSPA8 and TLQP-21. As evident from the electrostatic surface potential of HSPA8, the peptide binding region of the protein is highly charged and contains negatively charged residues. Specific electrostatic interactions between TLQP-21 and HSPA8 facilitate binding. Particularly, two positively charged residues Arg 10 and Arg 11 at the middle of the peptide and Arg 21 at the C-terminal region of the peptide TLQP-21 interacts strongly with the negatively charged region of the HSPA8 substrate binding site (Fig 7C).

## Discussion

Using affinity chromatography and mass spectrometry-based protein identification, we have identified cell surface-associated HSPA8 as a binding partner of the human VGF-derived peptide TLQP-21 in SH-SY5Y neuroblastoma cells. Cross-linking experiments further confirmed that TLQP-21 externally administered to live SH-SY5Y can be cross-linked to HSPA8, which strongly suggests that it binds a fraction of it located in the surface of the cells (Fig 3). In agreement with these results, DMR experiments confirmed binding of TLQP-21 to HSPA8 *in vitro*, with a K_d_ of ∼90 μM (Fig 4). This affinity is lower but overall relatively comparable to the affinity of HSPA8 for the phosphorylated peptide P140, which is of **∼7** μM [45]. P140 corresponds to a portion of the spliceosomal U1-70K snRNP protein, recognized by lupus CD4^+^ T cells. Upon binding, surface HSPA8 acts as a P140 peptide-presenting protein, inducing apoptosis of activated MRL/lpr CD4^+^ T cells via a specific mechanism that includes regulation of expression of inflammation-linked genes in CD4+ T cells [45]. It is noteworthy that P140 has successfully completed phase II clinical trials as a potential treatment of systemic lupus erythematosus [46–47].

Molecular modelling studies showed that TLQP-21 can be docked into the peptide binding pocket of HSPA8 (Fig 5). These studies show that TLQP-21 binds within the peptide binding site of HSPA8. The peptide interacts strongly with the twisted β-sheeted plate sheet region of the HSPA8 substrate binding site, with a tight spatial fit of the peptide inside the cavity. Molecular dynamics simulation reveals that the complex is highly stable throughout the simulation. Interestingly, during the simulation TLQP-21 comes closer towards the HSPA8 binding site and binds more tightly within the substrate binding pocket. Several hydrogen bonding interactions as well as strong electrostatic complementarity between TLQP-21 and HSPA8 stabilize the complex. An interesting observation from our simulation is that TLQP-21 is highly stable when bound to HSPA8 throughout the 100 ns simulation, evident from a highly stable RMSD and R_g_ profile (Fig 6A & 6B). Secondary structure analysis reveals that the peptide possesses strong helical propensity and its helical conformation is highly stable throughout the simulation. Particularly, the region 4–14 exists as α-helix when bound to the HSPA8 binding site. Interestingly, our recent simulation study of the free TLQP-21 peptide in solution reveals that the peptide is highly flexible in nature and fluctuates between many conformations during the simulation and its RMSD and R_g_ profile never stabilize throughout the simulation. The initial long helical conformation is not stable rather the peptide refolds in a more compact globular form. In agreement with our observation, a recent NMR study by Cero et al.[28], demonstrates that the murine TLQP-21 peptide adopts a well-defined α-helical conformation in the presence of cells expressing C3AR1, previously shown to be a receptor of this peptide; in contrast, in solution the peptide exhibits chemical shift index of a typical random coil conformation. It is noteworthy that HSPA8 shows higher binding affinity for short peptides containing basic sequences with at least KK, KR, or RR from a phage display peptide library [48], and TLQP-21 contains precisely an RR pair in its sequence.

HSPA8, a constitutively expressed protein, is an unusual member of the HSP70 (Heat shock protein 70) family. HSPA8 is localized in the cytoplasm and nucleus, but under certain circumstances, a sizeable fraction of it is also found associated to the cell membrane[42, 45, 49–51].HSPA8 is also detected in the extracellular space, where it is actively released from intact cells, either free or associated with exosomes [43]. In normal neurons, cell membrane associated HSPA8 is found in lipid rafts [51]. Lipid rafts are known to act as ‘sorting platforms’ for signal transduction complexes, and it has been hypothesized that HSPA8 might associate with these complexes to stabilize them and modulate signal transduction [43]. In fact, HSPA8 and other members of the extended HSP70 family have been recently termed “chaperokines” to underscore their function as initiators and modulators of signal cascades originating on the cell surface [43]. One relevant example of this is the association of HSPA8 with A1 adenosine receptors in the cell membrane to form functional modules [52].On the other hand, numerous studies have shown that cell surface HSPA8 contributes to cell recognition by immune cells, and important roles of HSPA8 and HSPs in general in the immune response have been demonstrated.

HSPA8 and other HSPs, are extraordinarily abundant in the cell membrane proteome of cancer cells. Shin et al., 2003[42] obtained a profile of the cell surface proteomes of A549 lung adenocarcinoma, LoVo colon adenocarcinoma, Sup-B15 acute lymphoblastic leukemia, ovarian tumor cells, and of particular relevance to the present discussion, SH-SY5Y neuroblastoma cells, and, unexpectedly, found that HSPs are among the most abundant proteins there. It is of note that these authors subjected viable, intact cells, to biotinylation using an N-hydroxysuccinimide ester of a biotin analog to cross-link biotin to them. Then they affinity-captured and purified the tagged proteins on monomeric avidin columns, similarly to our experimental approach, and identified the captured proteins by mass spectrometry. This fully agrees with the notion that these membrane-associated HSPs are fully accessible from the outside of the cells. HSPs might reach the cell surface as either by active transportation from their site of synthesis to maintain structural integrity among receptor complexes or accompanying misfolded proteins or peptide fragments out of the cytosol via a non-classic pathway[42]. On the other hand, it has been speculated that HSPs on the surface of cancer cells might act as “danger signals” and play a key role in the activation of the immune defensive response against tumor cells[53]. In this respect, as already mentioned, cell surface localized HSPA8 plays an active role in the immune mechanisms associated to systemic lupus erythematosus [45].

Considering all these facts, it is difficult and premature to conclude whether binding of TLQP-21 to cell surface HSPA8 has a positive or negative effect for the survival and proliferation of SH-SY5Y cells. On the one hand, it might result to activation of signals leading to proliferation, perhaps by activating some secondary membrane partner bound to HSPA8. This would be consistent with the general neurotrophic character of VGF and its derived peptides. Simultaneous ttargeting of HSPA8 and HSP1Ainducestumor-specific apoptosis[54], which highlights the important role of HSPA8 in viability of tumor cells.

Human TLQP-21 (TLQPP**SAL**RRRH**Y**HHALPP**S**R) shows extensive homology but also differences with mouse TLQP-21 (TLQPP**ASS**RRRH**F**HHALPP**A**R); Cero and coworkers[28] showed that human TLQP-21with a S20A substitution binds to human C3AR1; however, the binding was found to be substantially weaker in comparison to that of rodent TLQP-21. Moreover, human TLQP-21 was found to exert only a very limited biological activity, five times less than the corresponding rodent TLQP-21, upon interaction with rodent C3AR1 [27]. It is therefore likely that, although there might be some overlapping binding specificities, the main surface binding partners of human *vs*. murine TLQP-21, and by extension, VGF-derived bioactive peptides, are different. Cell type-derived differences cannot be ruled out, either.

We found that HSPA9/mortalin, another member of the HSP-70 family, also binds TLQP-21. HSPA9/mortalinis also abundantly present in the surface of cancer cells, including SH-SY5Y cells [42].Studies to elucidate its interaction with TLQP-21 are therefore warranted; in particular, to confirm if TLQP-21 binds to a cell surface fraction of HSPA9. If this were the case,it would suggest that TLQP-21 binds HSPs with a certain promiscuity, which would agree with the fact that many different fragments of VGF, with variable biological activities and potencies are dynamically generated, pointing to a signaling system with limited specificity.

The understanding of the molecular mechanisms and signaling events by which VGF-derived active peptides exert their many physiological actions is in its infancy. In what respects to our results, extensive work remains ahead before the consequences of the HSPA8–TLQP-21 partnership that we have unveiled can be fully understood and best exploited. Future work should aim to have a better understanding of the downstream consequences of the cell treatment with TLQP-21 which should uncover proteomic changes in TLQP-21 treated cells, and intracellular molecular mechanisms of TLQP-21 actions. At present, we do not know whether the interaction of TLQP-21 and HSPA8 that we have unveiled in SH-SY5Y is of relevance to cancer cells alone or to healthy cells, too. The downstream molecular consequences of TLQP-21 binding to HSPA8 should provide valuable information regarding the molecular mechanisms of TLQP-21 activity, and perhaps offer therapeutic strategies for pathologic conditions in which TLQP-21 is involved.
